# Screen printed passive components for flexible power electronics

**DOI:** 10.1038/srep15959

**Published:** 2015-10-30

**Authors:** Aminy E. Ostfeld, Igal Deckman, Abhinav M. Gaikwad, Claire M. Lochner, Ana C. Arias

**Affiliations:** 1 Department of Electrical Engineering and Computer Sciences, University of California, Berkeley, California 94720, USA

## Abstract

Additive and low-temperature printing processes enable the integration of diverse electronic devices, both power-supplying and power-consuming, on flexible substrates at low cost. Production of a complete electronic system from these devices, however, often requires power electronics to convert between the various operating voltages of the devices. Passive components—inductors, capacitors, and resistors—perform functions such as filtering, short-term energy storage, and voltage measurement, which are vital in power electronics and many other applications. In this paper, we present screen-printed inductors, capacitors, resistors and an RLC circuit on flexible plastic substrates, and report on the design process for minimization of inductor series resistance that enables their use in power electronics. Printed inductors and resistors are then incorporated into a step-up voltage regulator circuit. Organic light-emitting diodes and a flexible lithium ion battery are fabricated and the voltage regulator is used to power the diodes from the battery, demonstrating the potential of printed passive components to replace conventional surface-mount components in a DC-DC converter application.

Recent years have seen the development of a wide variety of flexible devices for applications in wearable and large-area electronics and the Internet of Things^1^,^2^. These include energy harvesting devices such as photovoltaics^3^, piezoelectrics^4^, and thermoelectrics^5^; energy storage devices such as batteries^6^,^7^; and power-consuming devices such as sensors^8^,^9^,^10^,^11^,^12^ and light sources^13^. While a great deal of progress has been made on the individual energy sources and loads, combining these components together into a complete electronic system typically also requires power electronics to overcome any mismatch between the source behavior and the loads’ requirements. For example, batteries produce a variable voltage dependent on their state of charge. If a load requires a constant voltage, or a higher voltage than the battery can produce, then power electronics are necessary. Power electronics use active components, transistors, to perform switching and control functions, as well as passive components—inductors, capacitors, and resistors. In a switching voltage regulator circuit, for example, inductors are employed to store energy during each switching cycle, capacitors are used to reduce voltage ripple, and the voltage measurement required for feedback control is accomplished using a resistor divider.

Power electronics appropriate for the demands of wearable devices such as the pulse oximeter^9^, which requires a few volts and a few milliamps, typically operate at frequencies in the range of hundreds of kHz to a few MHz, and require inductance and capacitance of several μH and several μF, respectively^14^. The conventional approach for manufacturing these circuits is to solder discrete components onto a rigid printed circuit board (PCB). While the active components of a power electronic circuit are often combined into a single silicon integrated circuit (IC), the passive components are usually external, either to allow customization of the circuit or because the required inductance and capacitance values are too large to be achieved in silicon.

Fabrication of electronic devices and circuits by additive printing processes offers a number of advantages in terms of simplicity and cost when compared to the conventional PCB-based manufacturing techniques. First, since many components of a circuit require the same materials, such as metal for contacts and interconnects, printing allows multiple components to be fabricated simultaneously, with relatively few processing steps and few sources of materials^15^. Replacing subtractive processes such as photolithography and etching with additive processes further reduces process complexity as well as materials waste^16^,^17^,^18^,^19^. In addition, the low temperatures used in printing are compatible with flexible and inexpensive plastic substrates, allowing large areas to be covered with electronics using high-speed roll-to-roll manufacturing processes^16^,^20^. For applications that cannot be fully realized using printed components, hybrid approaches have been developed in which surface-mount technology (SMT) components are attached at low temperature to flexible substrates alongside the printed components^21^,^22^,^23^. In such hybrid approaches, replacing as many SMT components as possible with their printed counterparts is still desirable to reap the benefits of the additive processes and improve the overall flexibility of the circuit. To achieve flexible power electronics, we propose a combination of SMT active components and screen-printed passive components, with particular emphasis on replacing bulky SMT inductors by planar spiral inductors. Of the various technologies for fabricating printed electronics, screen printing is especially well suited for passive components because of its large film thickness (which is necessary to minimize series resistance of metallic features) and its high printing speed, even when covering centimeter-scale areas with material^24^.

It is essential to minimize losses in passive components for power electronics, since the efficiency of the circuit directly affects the size of the energy source that is required to power a system. This is particularly challenging for printed inductors, which consist of long coils and are therefore susceptible to high series resistance. As a result, although there has been some effort toward minimizing resistance of printed coils^25^,^26^,^27^,^28^, there remains a lack of efficient printed passive components for power electronics. To date, many reported printed passive components on flexible substrates are designed to operate in resonant circuits for radio frequency identification (RFID) or energy harvesting purposes^10^,^12^,^25^,^27^,^28^,^29^,^30^,^31^. Others focus on materials or fabrication process development and demonstrate general-purpose components that are not optimized for a particular application^26^,^32^,^33^,^34^. Power electronic circuits such as voltage regulators, by contrast, tend to utilize larger components than the typical demonstrations of printed passives and do not require resonance, thus demanding different component designs.

Here, we present the design and optimization of screen-printed inductors in the μH range to achieve minimal series resistance and high performance at frequencies relevant to power electronics. Screen-printed inductors, capacitors, and resistors with various component values are fabricated on flexible plastic substrates. The suitability of these components for flexible electronics is first demonstrated in a simple RLC circuit. Printed inductors and resistors are then integrated with an IC to form a step-up voltage regulator. Finally, organic light-emitting diodes (OLEDs) and a flexible lithium-ion battery are fabricated, and the voltage regulator is used to power the OLEDs from the battery.

## Results

### Inductors

To design printed inductors for power electronics, we first predicted the inductance and DC resistance of a range of inductor geometries based on the current sheet model presented in Mohan *et al.*^35^, and fabricated inductors of different geometries to confirm the accuracy of the model. A circular shape was selected for the inductors in this work because higher inductance can be achieved with lower resistance compared to polygon geometries^36^. The effect of the type of ink and the number of print cycles on resistance was determined. These results were then used along with the current sheet model to design 4.7 μH and 7.8 μH inductors optimized for minimum DC resistance.

**Figure 1:**
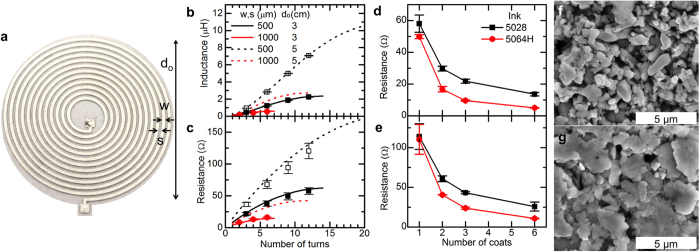
(**a**) Photograph of a screen-printed inductor, indicating geometric parameters. Diameter is 3 cm. Inductance (**b**) and DC resistance (**c**) for a variety of inductor geometries. Lines and markers correspond to calculated and measured values, respectively. (**d,e**) DC resistance of inductors L1 and L2, respectively, screen-printed from Dupont 5028 and 5064H silver inks. (**f,g**) SEM micrographs of films screen-printed from Dupont 5028 and 5064H, respectively.

The inductance and DC resistance of a spiral inductor can be described by a few parameters: the outer diameter d_o_, turn width w and spacing s, number of turns n, and the sheet resistance R_sheet_ of the conductor. Fig. 1a shows a photograph of a screen-printed circular inductor with n = 12, indicating the geometric parameters that determine its inductance. Inductance was calculated for a range of inductor geometries according to the current sheet model of Mohan *et al.*^35^, in which


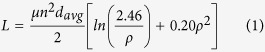


where μ is the permeability of the core (in this case, air); d_avg_ is the average diameter:


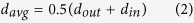


ρ is the fill ratio:


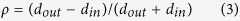


and d_in_ is the inner diameter:


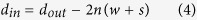


The DC resistance is given by


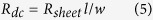


using the length l of the spiral:


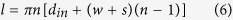


At high frequencies, the skin effect and parasitic capacitance change an inductor’s resistance and inductance from their DC values. It is desirable to operate the inductor at frequencies low enough that these effects are negligible and the device behaves as a constant inductance with a constant resistance in series. Thus, in this work we analyze the relationships between the geometric parameters, inductance and DC resistance, and use the results to obtain a given inductance with minimum DC resistance.

Inductance and resistance were calculated for a range of geometric parameters achievable with screen printing and expected to give inductances in the μH range. Outer diameters of 3 and 5 cm, line widths of 500 and 1000 μm, and various numbers of turns were compared. The calculations were performed assuming a sheet resistance of 47 mΩ/□, corresponding to a single 7 μm thick layer of Dupont 5028 silver microflake conductor printed using a 400-mesh screen, and setting w = s. Calculated inductance and resistance values are shown in Fig. 1b,c, respectively. The model predicts that inductance and resistance both increase as the outer diameter and number of turns are increased, or as the line width is decreased.

Inductors spanning a range of geometries and inductances were fabricated on polyethylene terephthalate (PET) substrates in order to assess the accuracy of the model predictions. Measured inductance and resistance values are shown in Fig. 1b,c. While the resistances show some deviation from the expected values, mainly due to variations in thickness and uniformity of the deposited ink, the inductance shows excellent agreement with the model.

These results can be used to design inductors having a desired inductance with minimum DC resistance. For example, suppose an inductance of 2 μH is desired. Figure 1b shows that this inductance can be achieved using 3 cm outer diameter, 500 μm line width, and 10 turns. The same inductance can also be produced using a 5 cm outer diameter, with either 500 μm line width and 5 turns or 1000 μm line width and 7 turns (also shown in the figure). Comparing the resistance of these three possible geometries in Fig. 1c reveals that the 5 cm inductor with 1000 μm line width has the lowest resistance of 34 Ω, about 40% lower than the other two. The generalized design process to achieve a given inductance with minimum resistance is summarized as follows: first, the largest allowable outer diameter is selected based on the spatial constraints imposed by the application. Then, the line width should be made as large as possible while still allowing the desired inductance to be reached, resulting in a high fill ratio (equation (3)).

Reducing the sheet resistance of the metal films, either by increasing the thickness or by using a material with higher conductivity, can further reduce the DC resistance without impacting the inductance. Two inductors, with geometric parameters given in Table 1, referred to as L1 and L2, were fabricated with varying number of coats to evaluate the change in resistance. As the number of coats of ink was increased, the resistance decreased proportionally as expected, as shown in Fig. 1d,e for inductors L1 and L2 respectively. Figure 1d,e show that up to 6-fold reduction in resistance can be achieved, through the application of 6 coats, while the greatest reduction in resistance (50–65%) occurs between 1 and 2 coats. A screen with a relatively small mesh size (400 threads per inch) was used to print these inductors because each coat of ink is relatively thin, allowing us to investigate the effect of conductor thickness on resistance. Similar thickness (and resistance) could be achieved faster by printing a smaller number of coats with a larger mesh size, as long as the patterned features remain larger than the minimum resolution of the mesh. This approach could be used to achieve the same DC resistance as the 6-coat inductors discussed here, but with higher production speed.

Figure 1d,e also show that a twofold reduction in resistance is achieved by using a higher-conductivity silver flake ink, Dupont 5064H. As seen in the SEM micrographs of films printed from the two inks, Fig. 1f,g, the lower conductivity of the 5028 ink arises from its smaller particle size and the presence of many voids between the particles in the printed film. The 5064H, on the other hand, has larger and more closely packed flakes, giving behavior closer to that of bulk silver. While this ink produced thinner films than the 5028 ink, 4 μm for a single coat and 22 μm for 6 coats, the enhancement in conductivity was substantial enough that the resistance was reduced overall.

Finally, while the inductance (equation (1)) depends on the period of the turns (w + s), the resistance (equation (5)) depends only on the line width w. Therefore, by increasing w relative to s, the resistance can be reduced even further. Two additional inductors, L3 and L4, were designed with w = 2s and large outer diameter, as shown in Table 1. These inductors were fabricated using 6 coats of Dupont 5064H, shown previously to give the highest performance. L3 had inductance of 4.720 ± 0.002 μH with resistance of 4.9 ± 0.1 Ω, while L4 had 7.839 ± 0.005 μH and 6.9 ± 0.1 Ω, in good agreement with the model predictions. This represents an improvement in the L/R ratio of more than an order of magnitude relative to the values in Fig. 1, due to the enhancements in thickness, conductivity, and w/s.

**Figure 2:**
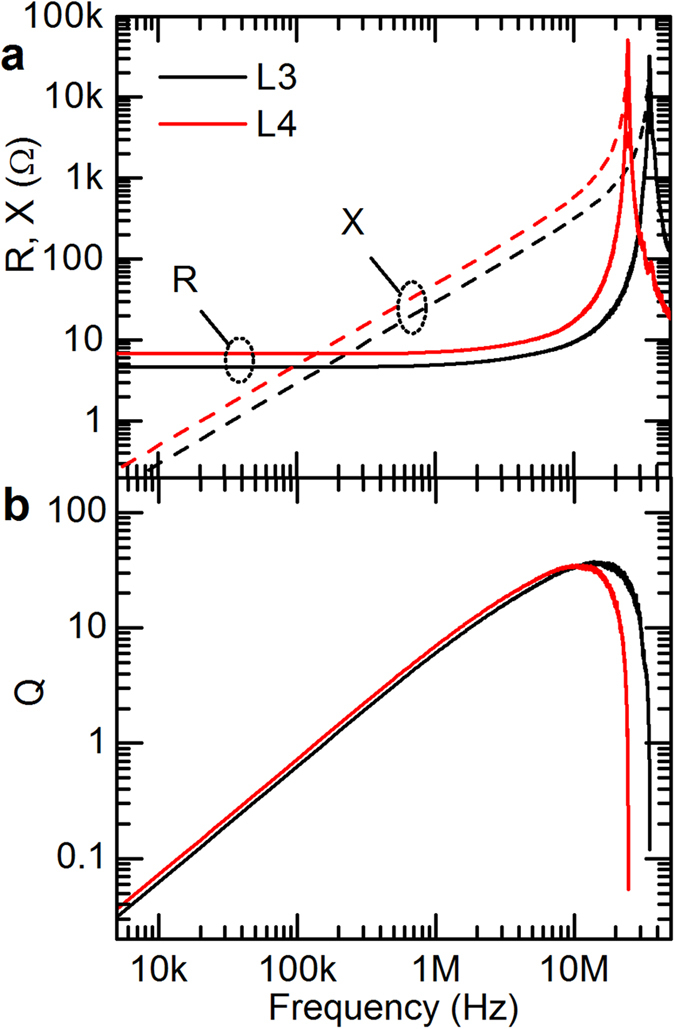
Measured resistance R and reactance X (**a**) and quality factor Q (**b**) versus frequency for inductors L3 and L4.

Although a low DC resistance is promising, assessing the suitability of the inductors for power electronics operating in the kHz-MHz range requires characterization at AC frequencies. Figure 2a shows the dependence of resistance and reactance of L3 and L4 on frequency. For frequencies below 10 MHz, the resistance stays roughly constant at its DC value, and the reactance increases linearly with frequency, implying a constant inductance as expected. The self-resonant frequency, defined as the frequency at which the impedance transitions from inductive to capacitive, occurs at 35.6 ± 0.3 MHz for L3 and 24.3 ± 0.6 MHz for L4. The dependence of the quality factor Q, equal to ωL/R, on frequency is shown in Fig. 2b. L3 and L4 reach their maximum quality factors of 35 ± 1 and 33 ± 1 at frequencies of 11 and 16 MHz respectively. The inductance of several μH and relatively high Q in the MHz frequencies make these inductors adequate replacements for conventional surface-mount inductors in low-power DC-DC converters.

### Capacitors

**Figure 3:**
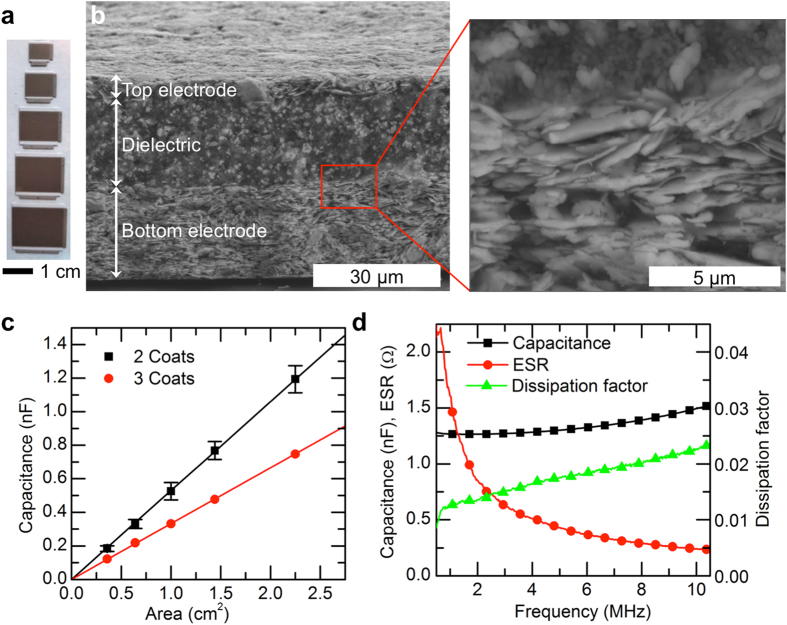
(**a**) Photographs of the capacitors with five different areas. (**b**) Cross-sectional SEM micrographs of a capacitor with two coats of dielectric, showing the barium titanate dielectric and silver electrodes. (**c**) Capacitance of capacitors with 2 and 3 coats of barium titanate dielectric and varying area, measured at 1 MHz. (**d**) Capacitance, ESR, and dissipation factor of a 2.25 cm^2^ capacitor with 2 coats of dielectric, vs. frequency.

To minimize the required footprint for a given capacitance, it is desirable to use a capacitor technology with a large specific capacitance, equal to the dielectric permittivity ε divided by the thickness of the dielectric. In this work, we chose a barium titanate composite for the dielectric, because it presents higher ε than other solution processed organic dielectrics. The dielectric layer was screen-printed between two layers of the silver conductor to form a metal-dielectric-metal structure. Capacitors with various dimensions on the centimeter scale, as shown in Fig. 3a, were fabricated using either two or three coats of dielectric ink, to maintain good yield. Figure 3b shows cross-sectional SEM micrographs of a representative capacitor fabricated with two coats of dielectric, for a total dielectric thickness of 21 μm. The top and bottom electrodes are one and six coats of 5064H, respectively. The micron-scale barium titanate particles are visible in the SEM image as brighter areas surrounded by the darker organic binder. The dielectric ink wets the bottom electrode well forming a clear interface with the printed metal film, as shown in the higher-magnification inset figure.

The capacitance scales proportionally with area as expected, as shown in Fig. 3c, with a specific capacitance of 0.53 nF/cm^2^ for two coats of dielectric and 0.33 nF/cm^2^ for three coats. These values correspond to a permittivity of 13. Capacitance and dissipation factor (DF) were also measured at varying frequency, as shown in Fig. 3d for a 2.25 cm^2^ capacitor with two coats of dielectric. We found that capacitance is relatively flat over the frequency range of interest, increasing by 20% from 1 to 10 MHz, while the DF increases from 0.013 to 0.023 over that same range. As the dissipation factor is a ratio of energy lost to energy stored per AC cycle, a DF of 0.02 signifies that 2% of the power handled by the capacitor is dissipated. This loss is also often expressed as a frequency-dependent equivalent series resistance (ESR), equal to DF/ωC, in series with the capacitor. As shown in Fig. 3d, the ESR is below 1.5 Ω for frequencies greater than 1 MHz and below 0.5 Ω for frequencies greater than 4 MHz. While the μF-scale capacitances needed for DC-DC converters would require prohibitively large areas using this capacitor technology, the 100 pF - nF capacitance range and low loss of these capacitors makes them suitable for other applications, such as filters and resonant circuits. A number of approaches could be used to increase the capacitance. A higher dielectric constant would increase the specific capacitance^37^; this can be achieved by increasing the concentration of barium titanate particles in the ink, for example. A smaller dielectric thickness could be used, although this would require a bottom electrode with lower roughness than the screen printed silver flakes. Thinner, lower roughness layers for capacitors can be deposited by inkjet printing^31^ or gravure printing^10^, which could be integrated with the screen printing process. Finally, multiple alternating layers of metal and dielectric could be printed in a stack and connected in parallel, increasing the capacitance per unit area^34^.

### Resistors

**Figure 4:**
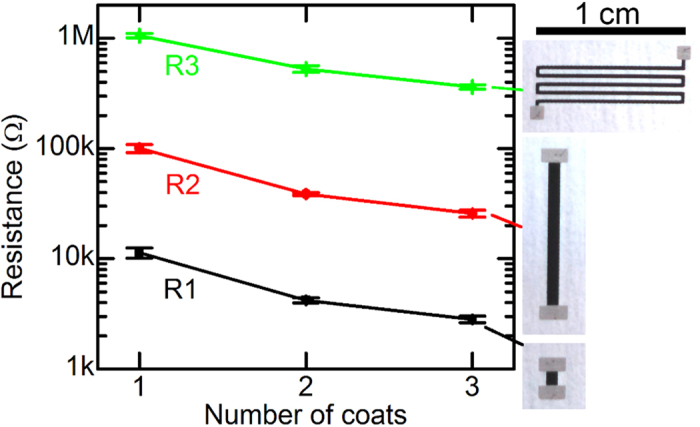
Resistance of three different resistor geometries with varying numbers of coats of carbon resistive ink. Photographs of the three resistors are shown on the right.

Voltage dividers, consisting of a pair of resistors, are typically used to perform the voltage measurement necessary for feedback control of a voltage regulator. For this type of application, printed resistors should present resistances in the range of kΩ-MΩ and low variation from device to device. Here, a single coat of screen-printed carbon ink was found to have a sheet resistance of 900 Ω/□. This information was used to design two straight-line resistors (R1 and R2) and one serpentine resistor (R3) with nominal resistances of 10 kΩ, 100 kΩ, and 1.5 MΩ, respectively. Resistances in between the nominal values were achieved by printing two or three coats of ink, as shown in Fig. 4 alongside photographs of the three resistors. 8–12 samples of each type were fabricated; in all cases, the standard deviation of the resistances was 10% or less. Samples with two or three coats tended to have slightly less variation in resistance than those with one coat. The small variation in measured resistance and close agreement with the nominal values suggests that other resistances in this range can be obtained straightforwardly by modifying the resistor geometry.

### RLC circuit

**Figure 5:**
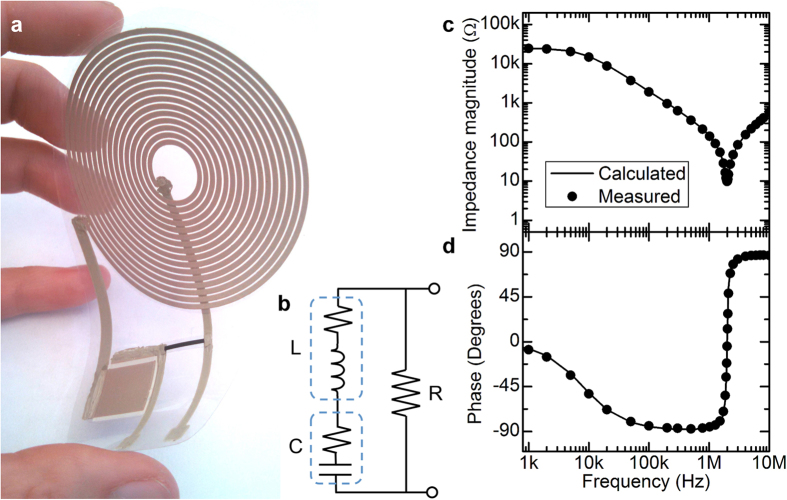
(**a**) Photograph of screen-printed RLC circuit using a series combination of 8 μH inductor and 0.8 nF capacitor, in parallel with a 25 kΩ resistor. (**b**) Model of the circuit including inductor and capacitor series resistances. (**c,d**) Impedance magnitude (**c**) and phase (**d**) of the circuit.

An RLC circuit, a classic textbook example of the combination of resistor, inductor, and capacitor, was fabricated to demonstrate and verify the behavior of the passive components integrated into a truly printed circuit. In this circuit, an 8 μH inductor and a 0.8 nF capacitor were connected in series, and a 25 kΩ resistor was placed in parallel with them. A photograph of the flexible circuit is shown in Fig. 5a. This particular series-parallel combination was selected because its behavior is dominated by each of the three components at different frequencies, allowing the performance of each one to be highlighted and assessed. The expected frequency response of the circuit was calculated, taking into account the 7 Ω series resistance of the inductor and the 1.3 Ω ESR of the capacitor. The circuit diagram is shown in Fig. 5b, and the calculated impedance magnitude and phase are shown in Fig. 5c and d along with measured values. At low frequency, the high impedance of the capacitor means that the behavior of the circuit is dominated by the 25 kΩ resistor. As the frequency increases, the impedance of the LC path decreases; the overall circuit behavior is capacitive until the resonant frequency of 2.0 MHz. Above the resonant frequency, the inductor impedance dominates. Figure 5 clearly shows the excellent agreement between the calculated and measured values over the entire frequency range. This signifies that the model used here, where the inductors and capacitors are ideal components with series resistances, is accurate for predicting circuit behavior at these frequencies.

### Voltage regulator

**Figure 6:**
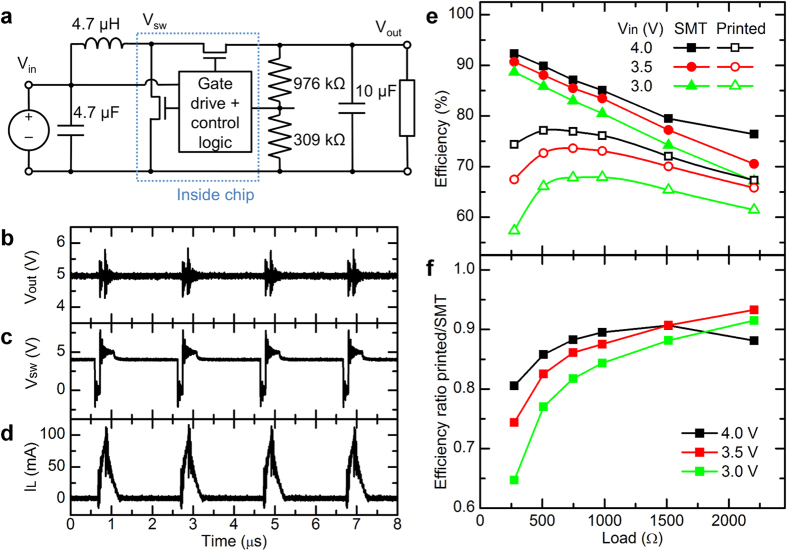
(**a**) Diagram of voltage regulator circuit. (**b–d**) Waveforms of (**b**) V_out_, (**c**) V_sw_ and (**d**) current into the inductor, with 4.0 V input voltage and 1 kΩ load resistance, measured using printed inductor. Surface-mount resistors and capacitors were used for this measurement. (**e**) Efficiency of a voltage regulator circuit using all surface-mount components vs. one with printed inductor and resistors, for various load resistances and input voltages. (**f**) Ratio of efficiencies of the surface-mount and printed circuits shown in (**e**).

Finally, the printed inductors and resistors were implemented in a step-up voltage regulator. The IC used in this demonstration was the Microchip MCP1640B^14^, a PWM-based synchronous boost regulator operating at 500 kHz. The circuit diagram is shown in Fig. 6a. A 4.7 μH inductor and two capacitors (4.7 μF and 10 μF) are used as the energy storage elements, and a pair of resistors is used to measure the output voltage for the feedback control. The resistor values were chosen to regulate the output voltage to 5 V. The circuit was fabricated on a PCB and its performance was measured over a range of load resistances and input voltages between 3 and 4 V, simulating the voltages of a lithium ion battery at various states of charge. The efficiency with printed inductors and resistors was compared to that with SMT inductor and resistors. SMT capacitors were used in all cases, because the capacitances required for this application were too large to accomplish using the printed capacitors.

Waveforms measured using a printed inductor are shown in Fig. 6b–d, for a 4.0 V input voltage and 1000 Ω load resistance. Figure 6c shows the voltage at the V_sw_ terminal of the IC; inductor voltage is V_in_-V_sw_. Figure 6d shows current into the inductor. Efficiency of the circuits with SMT and printed components is shown as a function of input voltage and load resistance in Fig. 6e, Fig. 6f shows the ratio of efficiency with printed components to that with SMT components. The measured efficiencies with the SMT components are similar to the expected values given in the manufacturer’s data sheet^14^. At high input currents (low load resistance and low input voltage), the efficiency is substantially lower with the printed inductor than the SMT inductor due to the higher series resistance. However, with higher input voltage and higher output current the resistive losses become less significant and the performance with the printed inductor begins to approach that of the SMT inductor. For load resistances >500 Ω with V_in_ = 4.0 V, or >750 Ω with V_in_ = 3.5 V, the efficiency with the printed inductor is >85% of the SMT inductor.

Comparing the current waveform in Fig. 6d with the measured power loss shows that resistive losses in the inductor are primarily responsible for the difference in efficiency between the printed and SMT circuits, as expected. The measured input and output power for 4.0 V input voltage and 1000 Ω load resistance were 30.4 mW and 25.8 mW for the circuit with SMT components, and 33.1 mW and 25.2 mW for the circuit with printed components, respectively. The loss in the printed circuit is therefore 7.9 mW, which is 3.4 mW higher than the circuit with SMT components. The RMS inductor current calculated from the waveform in Fig. 6d is 25.6 mA, giving an expected power loss of 3.2 mW due to its series resistance of 4.9 Ω. This is 96% of the measured 3.4 mW difference in DC power. Additionally, circuits were fabricated with a printed inductor and printed resistors as well as a printed inductor and SMT resistors, and no significant difference in efficiency was observed between them.

**Figure 7:**
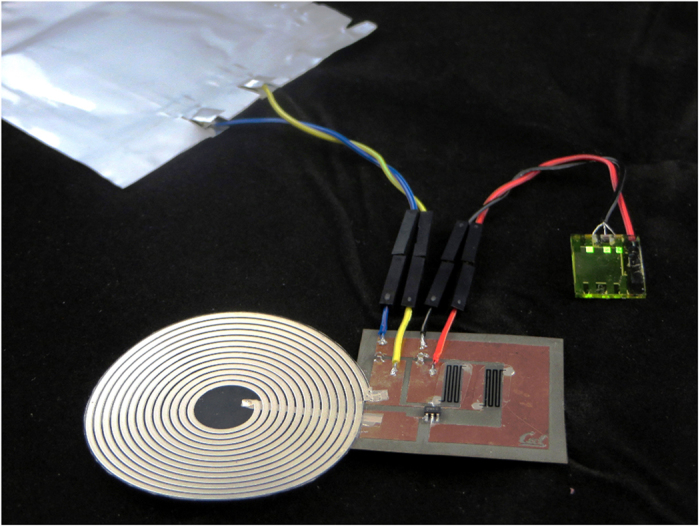
Photograph of a voltage regulator circuit on flex-PCB using printed inductor and resistors, using a flexible lithium ion battery to power three organic LEDs.

A voltage regulator was then fabricated on a flex-PCB (performance of this circuit with printed vs. SMT components is given in Supplementary Fig. S1) and connected between a flexible lithium-ion battery as the source and an array of OLEDs as the load. The OLEDs were fabricated according to Lochner *et al.*^9^, and each OLED pixel drew 0.6 mA at 5 V. The battery employed lithium cobalt oxide and graphite respectively as the cathode and anode, and was fabricated by blade coating, the most common battery printing method.^7^ The capacity of the battery was 16 mAh and its voltage was 4.0 V at the time of testing. Figure 7 shows a photograph of the circuit on a flex-PCB, powering three OLED pixels connected in parallel. This demonstration shows the potential of the printed power components to be integrated with other flexible and organic devices to form more complex electronic systems.

## Discussion

We have demonstrated screen-printed inductors, capacitors, and resistors with a range of values on flexible PET substrates, with the goal of replacing surface-mount components in power electronics. We have shown that the resistance of the inductors, which is of great concern for power electronics, can be reduced by more than an order of magnitude by designing the spiral with large diameter, fill ratio, and line width-space width ratio, and by using a thick layer of low resistivity ink. The components were integrated into a fully printed and flexible RLC circuit and show predictable electrical behavior in the kHz-MHz frequency range that is most of interest for power electronics.

A typical use case for printed power electronics would be in a wearable or product-integrated flexible electronic system powered by a flexible rechargeable battery, such as lithium ion, which produces a variable voltage depending on its state of charge. If the loads, which would include printed and organic electronic devices, require a constant voltage or one that is higher than the battery output, a voltage regulator is needed. For this reason, the printed inductor and resistors were integrated into a step-up voltage regulator alongside a conventional silicon IC, which was used to power OLEDs at a constant voltage of 5 V from a variable-voltage battery source. Efficiency of the circuit surpassed 85% of that of a control circuit using surface-mount inductor and resistors over a range of load currents and input voltages. Despite the material and geometry optimization, resistive losses in the inductor remained the limiting factor of the circuit performance at high current levels (input current greater than about 10 mA). At lower currents, however, the losses in the inductor were reduced and the overall performance became limited by the IC efficiency. Since many printed and organic devices require relatively low currents, such as the small OLEDs used in our demonstration, the printed power inductor can be deemed appropriate for this type of application. Higher overall converter efficiency may be achieved by utilizing an IC designed to have the highest efficiency at lower current levels.

In this work, voltage regulators were built upon conventional PCB, flex-PCB, and soldering techniques for the surface-mount components, and the printed components were fabricated on separate substrates. However, the low temperatures and high-viscosity inks used to produce screen-printed films should allow the passive components, as well as interconnects between devices and contact pads for surface-mount components, to be printed on arbitrary substrates. This combined with the use of existing low-temperature conductive adhesives for the surface-mount components would allow the entire circuit to be built, without subtractive processes such as PCB etching, on an inexpensive substrate such as PET. The screen-printed passive components developed in this work therefore help to pave the way for flexible electronic systems integrating energy sources and loads with high-performing power electronics, using inexpensive substrates, primarily additive processes, and a minimum number of surface-mount components.

## Methods

### Fabrication of screen printed passive components

All layers of the passive components were screen printed onto flexible PET substrates, 76 μm in thickness, using an Asys ASP01M screen printer and stainless steel screens supplied by Dynamesh Inc. Mesh size was 400 threads per inch for the metal layers and 250 threads per inch for the dielectric and resistor layers. Screen printing was performed using a squeegee force of 55 N, print speed of 60 mm/s, snap-off distance of 1.5 mm, and Serilor squeegees with hardness of 65 durometer (for metal and resistor layers) or 75 durometer (for dielectric layer).

Conductive layers—inductors and the contacts to the capacitors and resistors—were printed from either Dupont 5082 or Dupont 5064H silver micro-flake ink. Resistors were printed from Dupont 7082 carbon conductor. For the capacitor dielectric, Conductive Compounds BT-101 barium titanate dielectric was used. Each coat of dielectric was produced using a double pass (wet-wet) print cycle to improve uniformity of the film. For each component, the effect of multiple print cycles on component performance and variability was examined. Samples made with multiple coats of the same material were allowed to dry for 2 minutes at 70 °C between coats. After the final coat of each material, the samples were baked at 140 °C for 10 minutes to ensure complete drying. The screen printer’s automatic alignment feature was used to align subsequent layers. Contacts to the center of the inductor were made by cutting a via into the center pad and stencil printing a trace on the backside of the substrate with Dupont 5064H ink. Interconnects between printed devices were also stencil printed from Dupont 5064H. For the demonstration of printed components and SMT components together on a flex-PCB shown in Fig. 7, printed components were attached using Circuit Works CW2400 conductive epoxy and SMT components were attached using conventional soldering.

### Fabrication of lithium ion battery

Lithium cobalt oxide (LCO) and graphite based electrodes served as the cathode and anode for the battery, respectively. Slurry for the cathode was a mixture of 80 wt% LCO (MTI Corp.), 7.5 wt% graphite (KS6, Timcal), 2.5 wt% carbon black (Super P, Timcal) and 10 wt% polyvinylidene fluoride (PVDF, Kureha Corp.) and for the anode was a mixture of 84 wt% graphite, 4 wt% carbon black and 13 wt% PVDF. *N*-methyl-2-pyrrolidone (NMP, Sigma Aldrich) was used to dissolve the PVDF binder and disperse the slurry. The slurries were homogenized by stirring overnight with a vortex mixer. A 0.0005” thick stainless steel foil and 10 μm nickel foil served as the current collectors for the cathode and anode, respectively. The inks were printed on the current collector with a doctor blade at a print speed of 20 mm/s. The electrodes were heated in an oven at 80 °C for 2 hr to remove the solvent. The height of the electrode after drying was ~60 μm resulting in theoretical capacity of 1.65 mAh/cm^2^ based on the weight of the active material. The electrodes were cut to a dimension of 1.3 × 1.3 cm^2^ and heated overnight in a vacuum oven at 140 °C before sealing them with aluminum-laminated pouch in nitrogen filled glove box. Polypropylene based membrane separated with anode and cathode and a solution of 1M LiPF_6_ in EC/DEC (1:1) served as the electrolyte for the battery.

### Fabrication of OLEDs

Green OLEDs were fabricated from a blend of poly(9,9-dioctylfluorene-co-n-(4-butylphenyl)-diphenylamine) (TFB) and poly((9,9-dioctylfluorene-2,7-diyl)-alt-(2,1,3-benzothiadiazole-4, 8-diyl)) (F8BT), according to the procedure outlined in Lochner *et al.*^9^.

### Characterization

Film thickness was measured with a Dektak stylus profilometer. The films were cut to prepare cross-sectioned samples for a study by scanning electron microscope (SEM). A FEI Quanta 3D field emission gun (FEG) SEM was used to characterize the structure of the printed films and confirm thickness measurements. SEM study was carried out under 20 keV accelerating voltage and typical working distance of 10 mm.

DC resistances, voltages, and currents were measured with a digital multimeter. AC impedance of inductors, capacitors, and circuits was measured with an Agilent E4980 LCR meter for frequencies below 1 MHz and an Agilent E5061A network analyzer for frequencies above 500 kHz. Voltage regulator waveforms were measured with a Tektronix TDS 5034 oscilloscope.

## Additional Information

**How to cite this article**: Ostfeld, A. E. *et al.* Screen printed passive components for flexible power electronics. *Sci. Rep.* **5**, 15959; doi: 10.1038/srep15959 (2015).

## Supplementary Material

Supplementary Information

## Figures and Tables

**Table 1:** Geometric parameters and inductance of inductors fabricated in this work.

Inductor Name	Outer Diameter (cm)	Line Width (μm)	Space Width (μm)	Number of Turns	Inductance (μH)	
					Calculated	Measured
L1	3	500	500	12	2.3	2.23 ± 0.02
L2	5	500	500	12	7.2	7.06 ± 0.04
L3	5	1000	500	13	4.7	4.720 ± 0.002
L4	6	1000	500	16	8.0	7.839 ± 0.005
