# Supplementary material for: Screen printed passive components for flexible power electronics
Source: Sci Rep. 2015 Oct 30;5:15959. doi: 10.1038/srep15959 (PMC4626765; doi:10.1038/srep15959)
Supplement: Supplementary Information [file srep15959-s1.pdf]

Supplementary Information
Screen printed passive components for flexible power electronics

Aminy E. Ostfeld, Igal Deckman, Abhinav M. Gaikwad, Claire M. Lochner, and Ana C. Arias

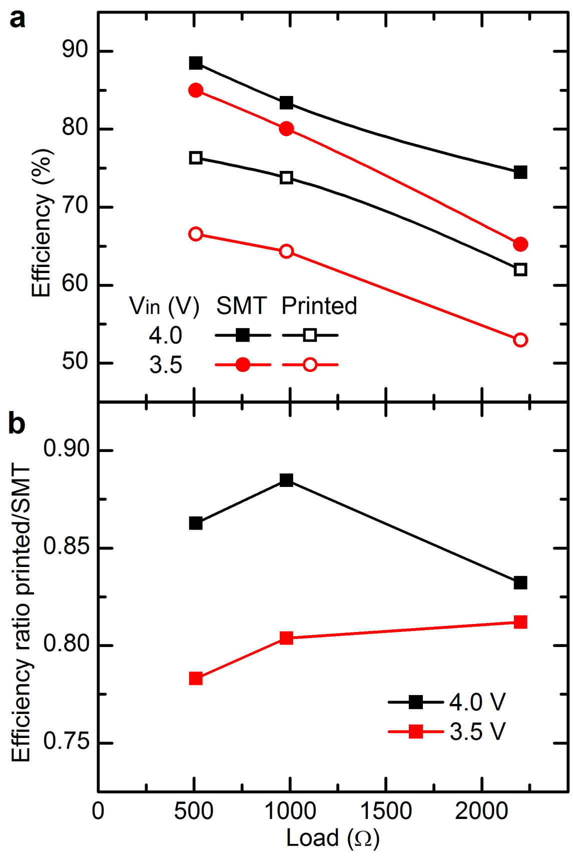

Figure S1. (a) Efficiency of a voltage regulator circuit on flex-PCB using all surface-mount (SMT) components vs. one with printed inductor and resistors, for various load resistances and input voltages. (b) Ratio of efficiencies of the surface-mount and printed circuits shown in (a).
